# Biological Differentiation of Dampness-Heat Syndromes in Chronic Hepatitis B: From Comparative MicroRNA Microarray Profiling to Biomarker Identification

**DOI:** 10.1155/2020/7234893

**Published:** 2020-01-19

**Authors:** Li Wen, Cen Jiang, Ting-Jun Wan, Dong Wang, Di Yan, Gui-Yu Li, Yue Su, Xi-Yang Liu, Li-Jun Rong, Hua Ye, Bai-Xue Li, Quan-Sheng Feng

**Affiliations:** ^1^College of Basic Medical Sciences, Chengdu University of Traditional Chinese Medicine, Chengdu 610075, China; ^2^Department of Microbiology and Immunology, College of Medicine, University of Illinois at Chicago, Chicago, IL 60612, USA; ^3^College of Medical Information Engineering, Chengdu University of Traditional Chinese Medicine, Chengdu 610075, China

## Abstract

Increasing interest is aroused by traditional Chinese medicine (TCM) treatment of chronic hepatitis B (CHB) based on specific TCM syndrome. As the most common CHB syndromes, spleen-stomach dampness-heat (SSDH) syndrome and liver-gallbladder dampness-heat (LGDH) syndrome are still apt to be confused in TCM diagnosis, greatly hindering the stable exertion of TCM effectiveness. It is urgently needed to provide objective and biological evidences for differentiation and identification of the two significant syndromes. In this study, microRNA (miRNA) microarray analyses coupled with bioinformatics were employed for comparative miRNA profiling of SSDH and LGDH patients. It was found that the two syndromes had both the same and different significantly differentially expressed miRNAs (SDE-miRNAs). Commonness and specificity were also both found between their SDE-miRNA-based bioinformatics analyses, including Hierarchical Clustering, Gene Ontology (GO), Kyoto Encyclopedia of Genes and Genomes (KEGG) pathways, and miRNA-GO/pathway networks. Furthermore, syndrome-specific SDE-miRNAs were identified as the potential biomarkers, including hsa-miR-1273g-3p and hsa-miR-4419b for SSDH as well as hsa-miR-129-1-3p and hsa-miR-129-2-3p for LGDH. All these laid biological and clinical bases for classification and diagnosis of the two significant CHB dampness-heat syndromes including SSDH and LGDH, providing more opportunities for better application of TCM efficacy and superiority in CHB treatment.

## 1. Introduction

Chronic hepatitis B (CHB) is a potentially life-threatening liver disease caused by hepatitis B virus (HBV) infection. It can progress to cirrhosis and hepatocellular carcinoma (HCC), leading to continuously increasing morbidity [1, 2]. It is estimated that 257 million people are living with HBV and Ca. 887 thousand people die annually from HBV-related disease worldwide [3]. These result in increasing healthcare cost and socioeconomic burdens [4]. CHB always represents a major global health challenge, and its prevention and control remains a significant issue.

Traditional Chinese medicine (TCM), with thousands of years of effective clinical practice, has become an important complementary and alternative medical system and aroused increased attention [5, 6]. It has been verified that TCM can relieve the clinical symptoms, reduce the liver injury, and slow the disease progression of CHB patients. [7–9]. TCM syndrome (also called “*ZHENG*”) is the basic concept of TCM theory. It describes special phenotype with comprehensive symptoms and signs of patients at a particular stage of disease [10]. The identification of TCM syndrome is the key to guide the specific TCM prescription [11, 12].

According to the standards of TCM syndrome differentiation for viral hepatitis [13], dampness-heat syndrome is the top popular CHB syndrome. In our previous study on CHB syndrome distribution (from 1260 CHB patients) [14], it was also found that dampness-heat syndrome occupied the largest proportion (46%) of the total CHB syndromes. All these indicated the significant importance of dampness-heat syndrome in CHB. Notably, spleen-stomach dampness-heat (SSDH) syndrome and liver-gallbladder dampness-heat (LGDH) syndrome are not only the most common dampness-heat syndromes but also the top two CHB syndromes, taking up 17% and 15%, respectively. However, it is still difficult to discriminate between SSDH and LGDH syndromes and apt to confuse their TCM treatments because they have some similar symptoms (also, see Supplementary Materials). Thus, the two dampness-heat syndromes were focused on in this study.

Currently, the classification of TCM syndromes is still debated [15] since the biological evidence remains lacking. In addition, syndrome differentiation currently depends on traditional observation, auscultation, interrogation, palpation, and the clinical experiences of TCM practitioners [16, 17]. Such diagnosis is usually accompanied with subjectivity, ambiguity, and nonrepeatability to some extent, which greatly impedes the stable exertion of TCM effectiveness and superiority [18]. This issue is particularly prominent for the identification of SSDH and LGDH syndromes. In traditional TCM diagnosis, SSDH and LGDH were classified by both the common and different symptoms (see the Supplementary Materials). Patients with SSDH syndrome usually present some special symptoms such as distending pain in lateral thorax, whereas LGDH patients are usually accompanied with ventosity, distention, and fullness. Meanwhile, SSDH and LGDH patients share the same symptoms such as yellow and slimy fur, nausea, and yellow urine, which may lead to doubt in their differences and confusion in discrimination. Thus, it is urgently needed to scientifically prove the syndrome classification and explore objective diagnosis for SSDH and LGDH syndromes.

MicroRNAs (miRNAs) are endogenous, noncoding, single-stranded RNAs of 19–25 nucleotides in length [19]. They play vital roles in regulating the global signaling networks and pathways involved in the pathological progression of liver diseases [20]. MiRNAs are attractive as potential biomarkers in recent years because they are specific to various disease states and reasonably stable under various environments [21]. Due to the advantages of high throughput, sensitivity, and accuracy [22], miRNA microarray profiling is regarded as a powerful strategy for demonstrating the expression levels of overall miRNAs [23]. It has become a predictive biomarker signature for detecting and distinguishing human diseases from healthy control (HC) over the past decade, such as colorectal cancer [24], chronic hepatitis C [25], and hepatocellular carcinoma [26]. Su et al. utilized circulating miRNAs to reveal the differences between two TCM syndromes in CHB [15, 27, 28], which inspired us to employ comparative miRNAs profiling to differentiate between the two CHB dampness-heat syndromes in this work.

We aim at biologically verifying the differentiation of SSDH and LGDH syndromes and identifying syndrome-specific miRNA biomarkers. Using miRNA microarray profiling, significantly differentially expressed miRNAs (SDE-miRNAs) of SSDH and LGDH patients were screened out firstly. These SDE-miRNAs were comparably analyzed by bioinformatics assays including Gene Ontology (GO) [29], Kyoto Encyclopedia of Genes and Genomes (KEGG) pathways [30], and miRNA-GO/pathway networks [31]. Thus the biological similarities and differences between SSDH and LGDH can be dissected and proved. Quantitative real-time polymerase chain reaction (qRT-PCR) was further conducted to measure the level of syndrome-specific SDE-miRNAs, so that potential diagnostic biomarkers for each syndrome can be validated.

## 2. Materials and Methods

### 2.1. Clinical Evaluation

Participants were satisfied with inclusion criteria including CHB diagnostic criteria of western medicine derived from “*Guidelines for Prevention and Treatment of Chronic Hepatitis B (December 2015)*” issued by Hepatology Branch and Infectious Disease Branch of China Medical Association. The diagnostic criteria for SSDH syndromes of CHB referred to the *Consensus of Experts in TCM Diagnosis and Treatment (2012)* as well as the research results and related monographs of the National Major Project of Science and Technology (no. 2012ZX10005001). The LGDH patients were diagnosed according to the *Guidelines for the Diagnosis and Treatment of CHB in Traditional Chinese Medicine (2018)* issued by Hepatobiliary Diseases Professional Committee of China Association of Traditional Chinese Medicine. The detailed contents of the inclusion criteria and exclusion criteria can also be seen in the Supplementary Materials.

### 2.2. Patients and Blood Collection

CHB patients with SSDH or LGDH syndromes were aged from 22 to 51 years and came from Chengdu Public Health and Medical Center. For both the miRNA microarray profiling and qRT-PCR analyses, peripheral blood of 15 SSDH patients, 15 LGDH patients, and 15 HC participants were collected in an EDTA anticoagulant tube. The plasma was collected from peripheral blood (4 mL) following the manufacturer's instructions. In brief, peripheral blood was centrifuged for 10 min at 1,700 g and 4°C. The supernatant was collected and centrifuged for 10 min at 2,000 g and 4°C. The supernatant was transferred into polypropylene tube as blood plasma sample and stored at −80°C.

### 2.3. RNA Extraction and miRNA Microarray Analysis

RNA isolation was performed on each blood plasma sample obtained as above. Total RNA was isolated by using Trizol, which was then followed by Qiagen miRNeasy Mini kit (Qiagen, Germany) according to the manufacturer's instructions. The isolated RNA was quantified with the spectrophotometer. The quality of isolated RNA was inspected by formaldehyde gel electrophoresis.

Genome-wide microRNA microarray profiling was performed using a human miRNA microarray platform (Agilent, USA). In short, 200 nanogram of miRNA was labelled using miRNA Complete Labeling and Hyb kit. Dried samples were placed into the hybridization oven overnight. After hybridization and washing, signals were measured by Agilent microarray scanner (G2565CA). The picture analysis and data extraction were processed with Agilent Feature Extraction software. The data were normalized by using Agilent Gene Spring software.

### 2.4. Bioinformatics Analysis

TargetScanHuman and miRTarBase were used for target gene prediction of the SDE-miRNAs. Hierarchical clustering analysis was performed with Cluster 3.0 software, which was originally written by Michael Eisen at Stanford University. GO and signaling pathway analysis were performed based on the DAVID Bioinformatics Resources 6.7 and KEGG database. The miRNA-target gene interactions, miRNA-GO networks and miRNA-pathway networks were analyzed by using Cytoscape software.

### 2.5. qRT-PCR for miRNA Verification

Significantly dysregulated miRNAs were validated by qRT-PCR. The miRNAs were separated by using miRcute serum/plasma miRNA extraction and separation kit (TIANGEN, China). RNA was reverse-transcribed to cDNA by employing cDNA synthesis kit (Exiqon, Denmark). qRT-PCR system (Eppendorf, Germany) combining with the predesigned primers (ABM, Canada) was used for miRNA quantification. For the reaction conditions, polymerase activation/denaturation was performed for 10 min at 95°C. 40 amplification cycles at 95°C for 10 seconds, 63°C for 15 seconds, and 72°C for 32 seconds were performed for miRNA quantification, followed by signal detection. The relative amount of miRNA was normalized against U6 snRNA (the internal control), and the fold change (FC) in the amount of each miRNA compared with the HC group was calculated by using the 2^−ΔΔCT^ method.

### 2.6. Statistical Analysis

The calculation of mean ± standard deviation (SD) and Students' *t* test was performed with GraphPad Prism 6.0 software. Only those miRNAs with FC >2 (or <0.5) and *P* < 0.05 compared with the HC group were considered as the SDE-miRNAs, and only these with all FC >1.5 (or <0.7) and *P* < 0.05 were considered as the potential biomarkers in the study.

## 3. Results

### 3.1. Patient Characteristics and miRNA Microarray Profiling

In order to gain biological insights into the similarities and differences between SSDH and LGDH syndromes, and further discover their miRNA biomarkers, 45 participants were included in our study, including 15 SSDH patients, 15 LGDH patients, and 15 HC volunteers. As shown in Supplementary [Supplementary-material supplementary-material-1], the sex and age were not significantly different between the three groups (*P* > 0.05). The distribution of ALT, AST, and TBIL were also found no significant differences between SSDH and LGDH groups (*P* > 0.05). The virus load in SSDH was higher than that in LGDH, but there was no significant difference (*P* > 0.05). The above clinical baseline data indicated that the participants were available for the next comparative miRNA microarray profiling.

According to the quantitative data of the identified miRNAs in microarray analysis, those with the FC > 2 (or<0.5) and *P* < 0.05 compared with the HC group were screened as the SDE-miRNAs, which were considered to be more valuable for further bioinformatics analysis [20]. Of these, 7 SDE-miRNAs were upregulated, and 3 SDE-miRNAs were downregulated in the SSDH group (Supplementary [Supplementary-material supplementary-material-1]), while 12 SDE-miRNAs were upregulated in the LGDH group (Supplementary [Supplementary-material supplementary-material-1]). Moreover, 4 of these SDE-miRNAs, namely, hsa-miR-122-5p, hsa-miR-320e, hsa-miR-1260a, and hsa-miR-483-3p, were shared by both SSDH and LGDH groups.

### 3.2. Hierarchical Clustering Analysis and Target Gene Prediction

**Figure 1:**
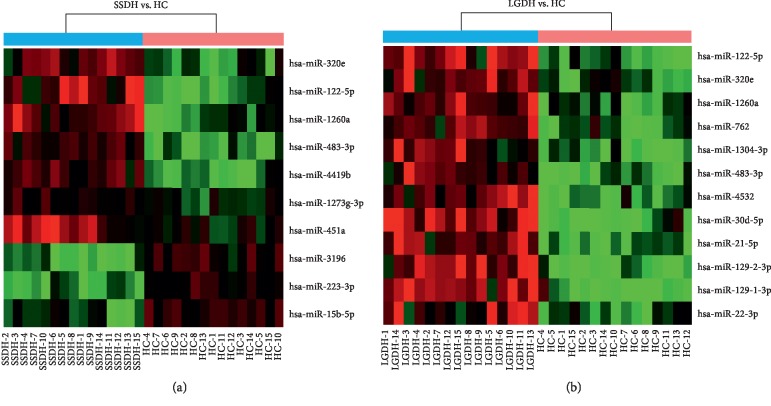
Hierarchical clustering analysis of the SDE-miRNAs in SSDH (a) and LGDH (b) syndromes. The red boxes and green boxes represent upregulation and downregulation of the corresponding SDE-miRNA, respectively.

To deeply dissect the biological commonalities and differences between SSDH and LGDH syndromes, intensive bioinformatics analysis of the SDE-miRNAs were performed. Firstly, the hierarchical clustering analysis [32] of SDE-miRNAs is shown in Figure 1. Then, the target genes of the SDE-miRNAs were predicted based on agreement between the databases of TargetScanHuman and miRTarBase [33]. A total of 1000 and 1200 target genes were predicted for the 10 SDE-miRNAs in SSDH and the 12 SDE-miRNAs in LGDH, respectively. To further show the regulatory relationships between SDE-miRNAs and their target genes, the biological interaction networks [34] were built (Supplementary [Supplementary-material supplementary-material-1]).

### 3.3. Comparative GO Annotation for SSDH and LGDH Syndromes

**Figure 2:**
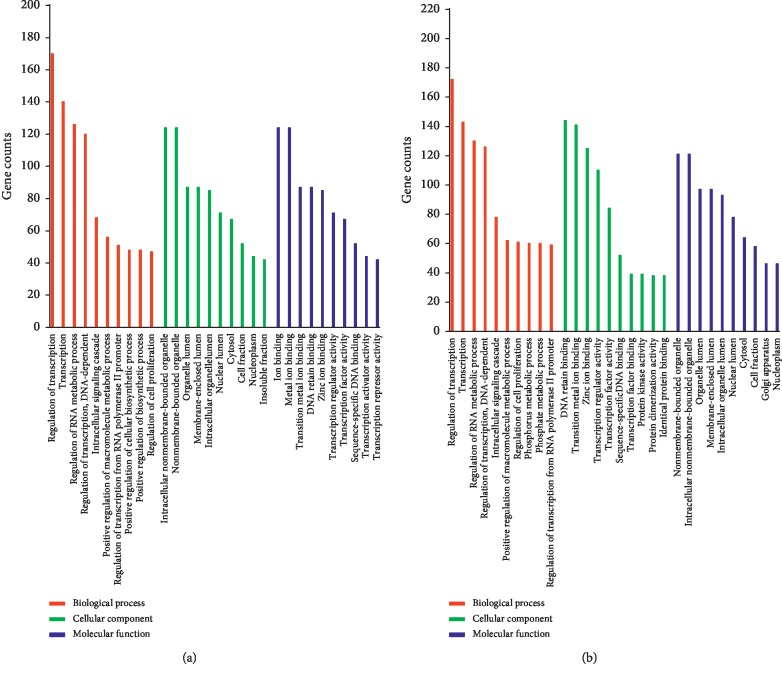
GO annotation of target genes of the SDE-miRNAs in SSDH (a) and LGDH (b) syndromes.

GO enrichment analysis were performed to determine the biological functions of the target genes of SDE-miRNAs. GO analysis mainly consists of three components: biological processes, cellular components, and molecular functions [29]. As shown in Figure 2, the top 10 terms of each GO component were plotted and compared between SSDH and LGDH syndromes. Notably, although the two dampness-heat syndromes shared some common terms in each component, there were some syndrome-specific terms. For biological processes (red histograms), positive regulation of cellular biosynthetic process (GO:0031328) and positive regulation of biosynthetic process (GO:0009891) were specific for SSDH, and whole phosphorus metabolic process (GO:0006793) and phosphate metabolic process (GO:0006796) were specifically related to LGDH syndrome. For cellular components (green histograms), the targets genes of the SDE-miRNAs in SSDH and LGDH syndromes were specifically involved with insoluble fraction (GO:0005626) and Golgi apparatus (GO:0005794), respectively. As for molecular functions (blue histograms), 4 terms were specific for each TCM syndromes, including ion binding (GO:0043167), metal ion binding (GO:0046872), transcription activator activity (GO:0016563) and transcription repressor activity (GO:0016564) for SSDH syndrome, as well as transcription factor binding (GO:0008134), protein kinase activity (GO:0004672), protein dimerization activity (GO:0046983), and identical protein binding (GO:0042802) for LGDH syndrome.

### 3.4. Comparative KEGG Pathway Analysis for SSDH and LGDH Syndromes

**Figure 3:**
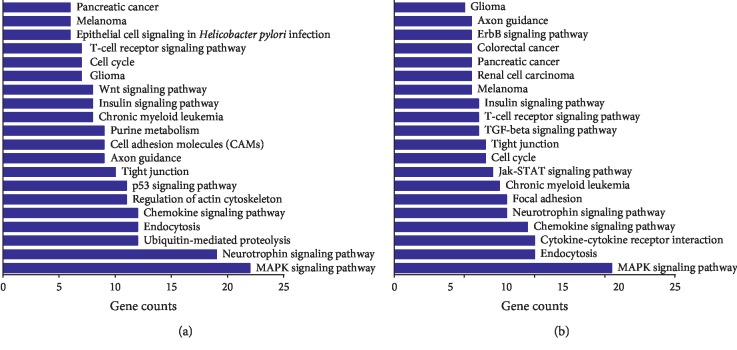
KEGG pathway analysis of target genes of the SDE-miRNAs in SSDH (a) and LGDH (b) syndromes.

KEGG pathway analysis [30] was carried out to further understand the functions and signaling pathways of the target genes. Twenty most commonly observed pathways were showed to compare the two syndromes (Figure 3). The result showed that the target genes of SDE-miRNAs in SSDH and LGDH group both mainly functioned in the MAPK signaling pathway (hsa04010), neurotrophin signaling pathway (hsa04722), chemokine signaling pathway (hsa04062), and endocytosis (hsa04144). Notably, the target genes in SSDH group specifically linked to 8 signaling pathways including ubiquitin-mediated proteolysis (hsa04120), p53 signaling pathway (hsa04115), regulation of actin cytoskeleton (hsa04810), cell adhesion molecules (CAMs) (hsa04514), purine metabolism (hsa00230), Wnt signaling pathway (hsa04310), glioma (hsa05214) and epithelial cell signaling in *Helicobacter pylori* infection (hsa05120). For LGDH syndrome, the target genes were also particularly involved in 8 signaling pathways including cytokine-cytokine receptor interaction (hsa04060), focal adhesion (hsa04510), Jak-STAT signaling pathway (hsa04630), TGF-beta signaling pathway (hsa04350), renal cell carcinoma (hsa05211), pancreatic cancer (hsa05212), colorectal cancer (hsa05210) and ErbB signaling pathway (hsa04012).

### 3.5. Comparative miRNA-GO/Pathway Network Analysis for SSDH and LGDH Syndromes

**Figure 4:**
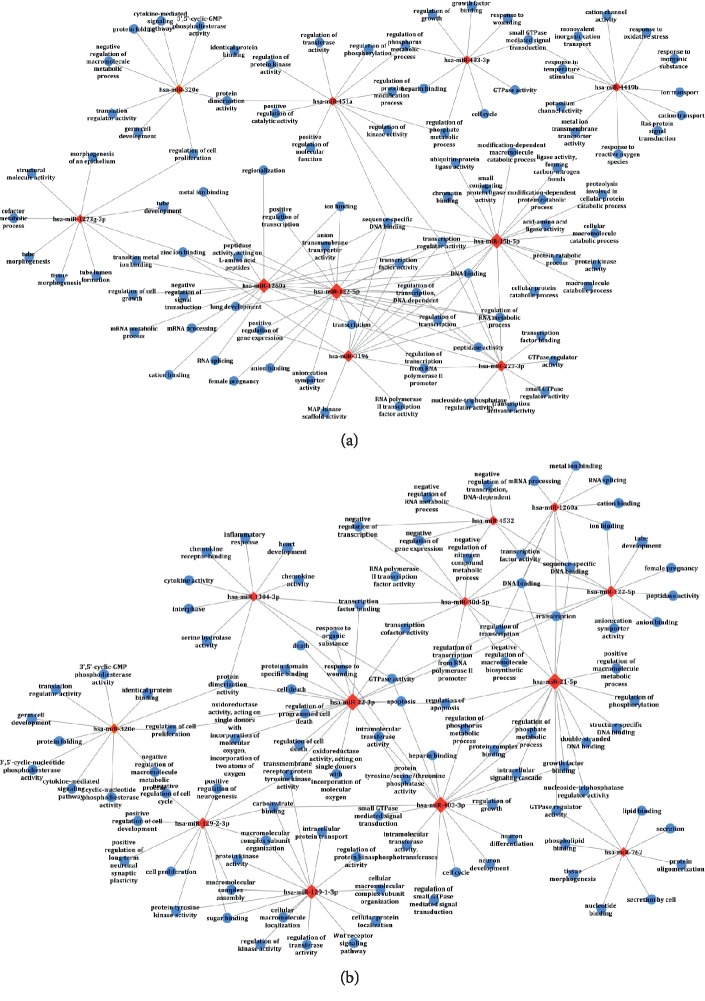
The miRNA-GO networks in the SSDH group (a) and LGDH group (b). The red squares (the central nodes) and the blue spots represent the SDE-miRNAs and the pathways, respectively. The lines represent interactions between the SDE-miRNA and the GO term.

**Figure 5:**
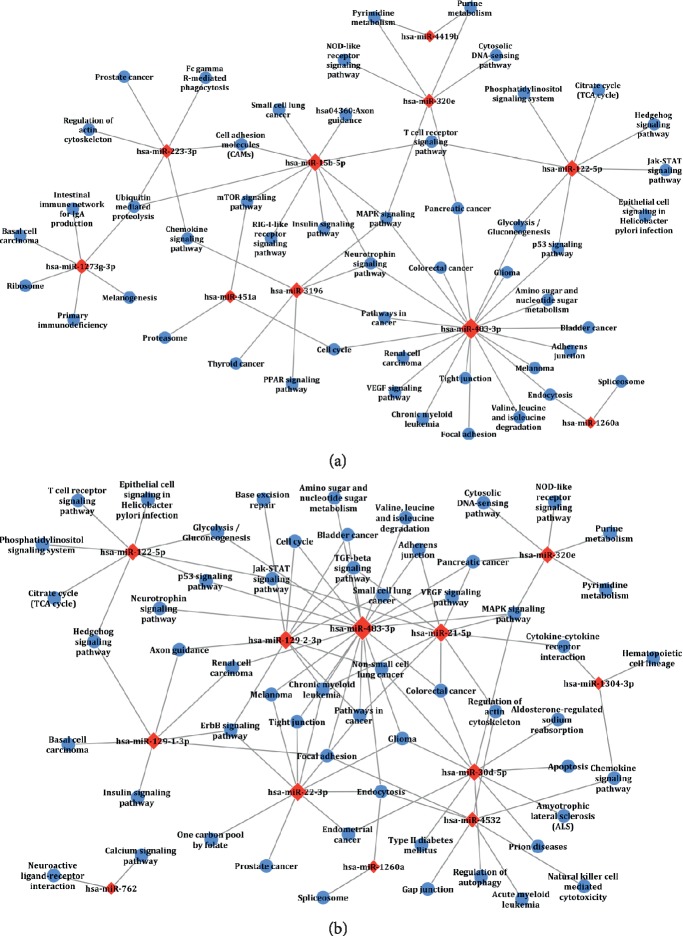
The miRNA-pathway networks in SSDH group (a) and LGDH group (b). The red squares (the central nodes) and the blue spots represent the SDE-miRNAs and their target gene pathways, respectively. The straight lines represent interactions between the SDE-miRNA and the pathways.

To further understand the association relationships between SDE-miRNAs and their corresponding GO terms and signaling pathways, miRNA-GO networks (Figure 4) and miRNA-pathway networks (Figure 5) were analyzed and demonstrated. As was shown in Figures 4 and 5, the SDE-miRNAs (the red squares) of SSDH or LGDH were all involved in the same network, suggesting that there were close biological correlations among the SDE-miRNAs of the syndrome. Furthermore, according to the study on biology network [35], the size of the red square indicates the complexity of regulatory relationship and the importance of the miRNA. For miRNA-GO networks, hsa-miR-122-5p, hsa-miR-1260a, hsa-miR-3196, and hsa-miR-15b-5p played crucial roles in modulating the molecular networks in SSDH syndrome (Figure 4(a)), whereas hsa-miR-483-3p, hsa-miR-22-3p, hsa-miR-21-5p, and hsa-miR-129-1-3p might be key regulators of pathogenesis in LGDH syndrome (Figure 4(b)). Moreover, the visualized miRNA-pathway networks indicated that hsa-miR-483-3p, hsa-miR-122-5p, hsa-miR-3196, and hsa-miR-15b-5p played prominent roles in signaling pathways related to SSDH syndrome (Figure 5(a)). Comparatively, hsa-miR-483-3p, hsa-miR-21-5p, hsa-miR-129-2-3p, and hsa-miR-22-3p carried considerable weight in LGDH groups (Figure 5(b)).

### 3.6. Validation of the Biomarkers for SSDH and LGDH Syndromes

**Figure 6:**
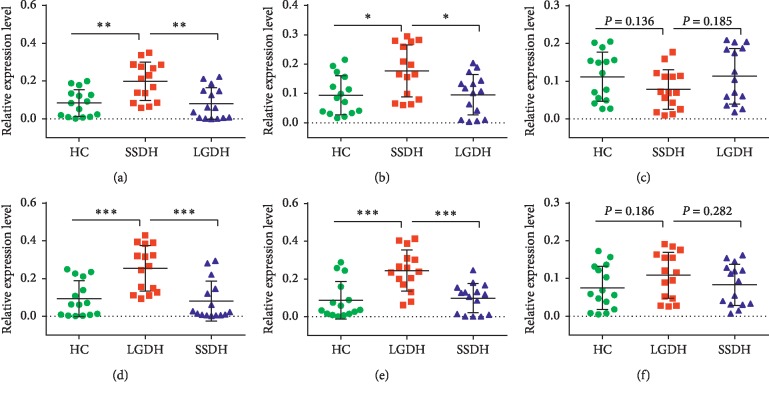
Validation of the biomarkers for SSDH and LGDH syndromes by qRT-PCR. Statistical difference analysis of the relative expression levels of hsa-miR-1273g-3p (a), hsa-miR-4419b (b), and hsa-miR-3196 (c) for SSDH compared with LGDH and HC, and hsa-miR-129-1-3p (d), hsa-miR-129-2-3p (e), and hsa-miR-21-5p (f) for LGDH compared with SSDH and HC (*n* = 15). ^*∗*^*P* < 0.05, ^*∗∗*^*P* < 0.01, ^*∗∗∗*^*P* < 0.001.

To validate the microarray results and further identify potential biomarkers for confirming and distinguishing between SSDH and LGDH syndromes in CHB, syndrome-specific SDE-miRNAs were randomly selected for qRT-PCR analysis. As shown in Figure 6, the expression level of 6 SDE-miRNAs was measured. To gain more reliability for syndrome-specificity, the expression level of each SDE-miRNA in one syndrome must suffer the comparison with that in the other syndrome and that in the HC group simultaneously. Only the SDE-miRNAs with both FC values >1.5 (or <0.7) and both *P* values <0.05 can be considered as biomarkers. For example, the expression level of hsa-miR-1273g-3p in SSDH group was significantly higher than both that in the HC group (FC = 2.12, *P* < 0.01) and that in the LGDH group (FC = 2.45, *P* < 0.01) (Figure 6(a)). Likewise, hsa-miR-4419b was significantly highly expressed in the SSDH group when compared with both HC and LGDH groups, with FC values of 1.88, 1.85 and *P* value was both lower than 0.05 (Figure 6(b)). For LGDH group, a significant hsa-miR-129-1-3p increase was shown when compared with the HC group (FC = 2.71, *P* < 0.001) and SSDH group (FC = 3.12, *P* < 0.001) (Figure 6(d)). Hsa-miR-129-2-3p was significantly overexpressed in LGDH group when compared with HC and SSDH groups, with FC values of 2.79, 2,49 and both *P* values lower than 0.001 (Figure 6(e)). These results are also in accordance with the microarray data. Thus, it was believed that hsa-miR-1273g-3p and hsa-miR-4419b could be employed as SSDH biomarkers and that hsa-miR-129-1-3p and hsa-miR-129-2-3p were available as LGDH biomarkers. However, hsa-miR-3196 (Figure 6(c)) and hsa-miR-21-5p (Figure 6(f)) with *P* > 0.05 were excluded as biomarkers.

## 4. Discussion

TCM has been proved effective for CHB treatment in practice for a long time. However, biological basis for classification and diagnosis of TCM syndromes of CHB are still lacking. Dampness-heat syndrome occupies the largest proportion of the total CHB syndromes. The top two dampness-heat syndromes, SSDH and LGDH are apt to be confused since they have some similar symptoms. Thus, the two syndromes were investigated in this study. Based on the microarray profiling data, the SDE-miRNAs including hsa-miR-122-5p, hsa-miR-320e, hsa-miR-1260a, and hsa-miR-483-3p might be related with the progression of CHB dampness-heat syndrome, as they were identified to be significant in both SSDH and LGDH groups. Hsa-miR-1273g-3p, hsa-miR-4419b, hsa-miR-451a hsa-miR-3196, hsa-miR-223-3p, and hsa-miR-15b-5p might be specifically linked to SSDH syndrome. Meanwhile, hsa-miR-129-1-3p, hsa-miR-129-2-3p, hsa-miR-21-5p, hsa-miR-1304-3p hsa-miR-30d-5p, hsa-miR-762, hsa-miR-4532, and hsa-miR-22-3p might play an important and special role in LGDH syndrome. These results indicated that both syndrome-common and syndrome-specific mechanisms may exist between SSDH and LGDH.

Most of the SDE-miRNAs have already been reported in previous virus hepatopathy-related studies. In general, miRNA could act as a cellular antiviral defense or help viruses establish a favorable environment for their replication and survival [20]. Hsa-miR-122 is a liver-specific miRNA and associated with the immune control of chronic HBV infection [36]. It has been found that hsa-miR-122-5p was upregulated during different phases of chronic HBV infection [37], which is in accordance with our microarray results. Hsa-miR-483-3p was differentially expressed in peripheral blood mononuclear cell from the chronic asymptomatic carriers [38] and upregulated in HBV-associated HCC [39]. Hsa-miR-320 was identified as the miRNA whose expression levels were altered by hepatitis virus infection [40] and showed downregulation in hepatitis B patients [41]. It has been demonstrated that hsa-miR-451a was differentially expressed in CHB and might play a crucial role in global signaling networks and pathways involved in CHB pathogenesis [20]. Hsa-miR-15b has been reported to be important during HBV infection because it could promote HBV replication by augmenting HBV enhancer I activity *via* directly targeting hepatocyte nuclear factor 1*α* [42]. Hsa-miR-1273g-3p might affect the activation and apoptosis of HSCs by targeting PTEN in hepatitis virus [43]. Hsa-miR-223-3p was found to be significantly dysregulated in HBV-positive patients [44]. It was further confirmed as a novel noninvasive biomarker of HBV-positive HCC at a very early stage of liver disease [45]. Hsa-miR-22 played a prominent role in HBV-related diseases [46], as it might be involved in HBV infection [47]. Hsa-miR-22-3p was found differentially expressed during different pathologic processes of CHB [20]. It has been indicated that hsa-miR-21-5p participated in inflammatory responses and hepatocyte proliferation and was closely related with liver disease [48–50]. Serum hsa-miR-21-5p was significantly elevated in CHB patients [51], which is consistent with our results. Hsa-miR-30d was significantly overexpressed in HBV-associated HCC patients [52], which was in accordance with the upregulation of hsa-miR-30d in our study. Khairy et al. have demonstrated that there was significant fold change of hsa-miR-129 in HCC patients compared with the HC group [53]. Hsa-miR-129-2 has been evaluated as a potential early diagnostic biomarker for HBV-related HCC since 85% of HCC patients at stage I could be distinguished by their miR-129-2 methylation levels [54]. Hsa-miR-4532 has been identified as a vital miRNA demonstrating a strong expressional response to HBV and could be used as an early diagnostic biomarker of hepatitis B [41]. All these above suggested that most of the identified SDE-miRNAs were biologically related to virus hepatopathy, and thus the microarray results were further validated. Some of the SDE-miRNAs (such as hsa-miR-1260a, hsa-miR-3196, and hsa-miR-762) have not been reported in virus hepatopathy-related studies, indicating that the finding in our research is novel and needs further investigation.

Detailed dissection of the identified SDE-miRNAs and the regulation mechanisms is of great significance for further understanding the pathogenesis and biological differences of SSDH and LGDH syndromes in CHB. Thus, intensive bioinformatics analysis based on the predicted target genes of SDE-miRNAs was carried out. According to GO data (Figure 2), the two dampness-heat syndromes shared some common GO terms, and they also owned syndrome-specific terms. KEGG pathway analysis (Figure 3) demonstrated that the target genes of SDE-miRNAs in the two syndromes both mainly functioned in the MAPK signaling pathway, neurotrophin signaling pathway, chemokine signaling pathway, and endocytosis. Meanwhile, there were also some syndrome-specific signaling pathways, such as ubiquitin-mediated proteolysis for SSDH syndrome and cytokine-cytokine receptor interaction for LGDH syndrome. Finally, the association relationship and the importance of the SDE-miRNAs in each TCM syndrome were determined by setting up miRNA-GO and miRNA-pathway networks (Figures 4 and 5). For SSDH and LGDH groups, although there were the same SDE-miRNAs (such as hsa-miR-483-3p and hsa-miR-122-5p) that played crucial roles in the networks, most of the key SDE-miRNAs in the networks of the two syndromes were different. All these above further validated that both biological similarity and differences existed between SSDH and LGDH syndromes. The results provided more evidence for the TCM practices, in which the syndromes are based on the same disease and discriminated by different symptoms of patients [11], and in which the prescription of TCM medications is determined by both the disease and the specific symptoms.

Furthermore, in order to provide more biological bases for objective diagnosis and accurate treatment of the two dampness-heat syndromes, qRT-PCR were experimented to identify potential biomarkers based on the syndrome-specific SDE-miRNAs. Comparisons among the three groups (SSDH, LGDH, and HC) were carried out. Only those with all FC >1.5 (or <0.7) and *P* < 0.05 were considered, and thus more reliability for syndrome-specificity was provided. Taken together, 4 SDE-miRNAs were validated as the diagnostic biomarkers, including hsa-miR-1273g-3p and hsa-miR-4419b for SSDH as well as hsa-miR-129-1-3p and hsa-miR-129-2-3p for LGDH.

However, some limitations also exist in this work. In order to enhance the value of this study in future, a large number of CHB patients with each syndrome should be further enrolled and tested. The importance of the potential diagnostic biomarkers of SSDH and LGDH syndromes should be further confirmed. Studies on molecular regulating mechanisms of the identified SDE-miRNAs in each syndrome need to be carried out, so that the pathogenesis of dampness-heat syndrome in CHB and therapeutic targets for treatment can be deeply elucidated. Moreover, other dampness-heat syndromes should also be investigated to comprehensively understand the dampness-heat syndrome of CHB.

## 5. Conclusion

By utilizing miRNA array profiling, 10 and 12 SDE-miRNAs were identified in SSDH and LGDH syndromes of CHB, respectively. Among these SDE-miRNAs, 4 were found in both dampness-heat syndromes, but 6 and 8 specifically linked to SSDH and LGDH, respectively. These results indicated that both biological similarity and syndrome-specificity existed in SSDH and LGDH patients. This was further confirmed by bioinformatics analyses, in which both same and different GO terms, KEGG pathways, and miRNA-GO/pathway networks were found between the two syndromes. Furthermore, syndrome-specific SDE-miRNAs were identified as the potential biomarkers, including hsa-miR-1273g-3p and hsa-miR-4419b for SSDH and hsa-miR-129-1-3p and hsa-miR-129-2-3p for LGDH, respectively. All these laid scientific basis for the differentiation and diagnosis of the two significant dampness-heat syndromes in CHB, providing more opportunities for stable exertion and better application of the efficacy and superiority of TCM in CHB treatment.

## Data Availability

The data used to support the findings of this study are available from the corresponding author upon request.

## Supplementary Materials

Supplementary Materials Table S1: clinical diagnosis of patients who participated in this study. Table S2: the SDE-miRNAs in SSDH patients. Table S3: the SDE-miRNAs in LGDH patients. Figure S1: the miRNA-target gene networks built using the target genes of the SDE-miRNAs in SSDH (a) and LGDH (b). The red squares (the central nodes) represent SDE-miRNAs, and the blue spots represent the target genes. The lines indicate the interactions between SDE-miRNAs and the target genes.
