# Supplementary material for: Biological Differentiation of Dampness-Heat Syndromes in Chronic Hepatitis B: From Comparative MicroRNA Microarray Profiling to Biomarker Identification
Source: Evid Based Complement Alternat Med. 2020 Jan 19;2020:7234893. doi: 10.1155/2020/7234893 (PMC6995329; doi:10.1155/2020/7234893)
Supplement: Supplementary Materials — Table S1: clinical diagnosis of patients who participated in this study. Table S2: the SDE-miRNAs in SSDH patients. Table S3: the SDE-miRNAs in LGDH patients. Figure S1: the miRNA-target gene networks built using the target genes of the SDE-miRNAs in SSDH (a) and LGDH (b). The red squares (the central nodes) represent SDE-miRNAs, and the blue spots represent the target genes. The lines indicate the interactions between SDE-miRNAs and the target genes. [file 7234893.f1.docx]

**Supplementary Materials**

**Biological Differentiation of Dampness-Heat Syndromes in Chronic Hepatitis B: From Comparative MicroRNA Microarray Profiling to Biomarker Identification**

Li Wen,^1^ Cen Jiang,^1^ Ting-Jun Wan,^1^ Dong Wang,^1^ Di Yan,^1^ Gui-Yu Li^1^, Yue Su,^1^ Xi-Yang Liu,^1^ Li-Jun Rong,^2^ Hua Ye,^3^ Bai-Xue Li,^1^ and Quan-Sheng Feng^1^

^1^ College of Basic Medical Sciences, Chengdu University of Traditional Chinese Medicine, Chengdu 610075, China
^2^ Department of Microbiology and Immunology, College of Medicine, University of Illinois at Chicago, Chicago, IL 60612, USA
^3^ College of Medical Information Engineering, Chengdu University of Traditional Chinese Medicine, Chengdu 610075, China

Correspondence should be addressed to Quan-Sheng Feng; [fengqs@163.com](mailto:fengqs@163.com), Bai-Xue Li; [baixuelee@163.com](mailto:baixuelee@163.com) and Hua Ye; [yehua@cdutcm.edu.cn](mailto:yehua@cdutcm.edu.cn).

**1.** **Diagnostic and inclusion criteria**

1. **Inclusion criteria**

CHB diagnostic criteria of western medicine (listed as follows) are derived from ‘*Guidelines for Prevention and Treatment of Chronic Hepatitis B (December in 2015)*’ issued by hepatology Branch and Infectious Disease Branch of China Medical Association.

i. With positive HBsAg for more than six months

ii. With positive HBV DNA viral load

iii. With persistent or intermittent elevation in ALT/AST levels or liver biopsy showing chronic hepatitis

All the enrolled patients must satisfy both the above CHB diagnostic criteria and following criteria:

i. Aged between 18-60 years old

ii. Signed informed consent for participation

1. **Diagnosis criteria for** **SSDH** **syndromes of CHB**

The diagnostic criteria for SSDH syndromes of CHB (listed as follows) referred to the *Consensus of Experts in TCM Diagnosis and Treatment* *(2012)* as well as the research results and related monographs of the National Major Project of Science and Technology (No. 2012ZX10005001).

The primary symptoms are:

i. Ventosity, distention and fullness

ii. Yellow and greasy tongue coating

The secondary symptoms are:

i. Nausea, loathing greasy and anorexia

ii. The body and eyes appear bright yellow, or the urine is yellow

iii. Sticky and smelly stool

The person who satisfies the following conditions can be diagnosed as CHB SSDH patient.

i. The person with all the primary symptoms

ii. The person with primary symptom ii and two of the secondary symptoms

iii. The person with primary symptom i as well as secondary symptoms i and ii

1. **Diagnosis criteria for LGDH syndromes of CHB**

The diagnostic criteria for LGDH syndromes of CHB (listed as follows) referred to the *Guidelines for the Diagnosis and Treatment of CHB in Traditional Chinese Medicine (2018)* issued by Hepatobiliary Diseases Professional Committee of China Association of Traditional Chinese Medicine.

The primary symptoms are:

i. Distending pain in lateral thorax

ii. Yellow and greasy tongue coating

The secondary symptoms are:

i. Anorexia, nausea and vomiting, loathing greasy

ii. Yellow urine

iii. Yellowing of the body and eyes

The person who satisfies the following conditions can be diagnosed as CHB LGDH patient.

i. The person with all the primary symptoms

ii. The person with primary symptom i and two of the secondary symptoms

iii. The person with primary symptom ii as well as secondary symptoms i and ii

**2. Exclusion criteria**

Patients were filtered once they satisfied one of the following criteria: pregnant women or nursing mothers; with cirrhosis or malignant tumor; with other types of hepatitis; with HIV infection; with drug-induced or toxic hepatic damage; with autoimmune hepatitis; with hereditary and metabolic liver disease; with other systemic diseases such as hypertension, diabetes mellitus and nephropathy; with unconsciousness, dementia or mental disease; family members disagree.

**3. Clinical diagnosis of patients who participated in this study**

As shown in Table S1, for HC group, SSDH patients and LGDH patients, the gender, age were not significantly different (*P* > 0.05). For SSDH and LGDH patients, the biochemical indexes such as ALT, AST, and TBIL were not significantly different (*P* > 0.05).

**Table S1.** Clinical diagnosis of patients who participated in this study

|  | Unit | SSDH (n=15) | LGDH (n=15) | HC (n=15) | *P* |
| --- | --- | --- | --- | --- | --- |
| Sex (female/male) |  | 2/8 | 3/7 | 4/6 | 0.497^※^ |
| Age (Mean ± SD) | years | 35.80±11.26 | 35.40±8.31 | 27.37±1.11 | 0.96^※^ |
| ALT (Mean ± SD) | IU/L | 390.50±211.65 | 312.40±240.76 |  | 0.93 |
| AST (Mean ± SD) | IU/L | 266.10±185.96 | 255.40±222.24 |  | 0.97 |
| TBIL (Mean ± SD) | *μ*mol/L | 27.37±11.63 | 28.20±8.34 |  | 0.74 |
| HBV DNA  (Mean ± SD) | 10^6 copies/mL | 29.5±10.5 | 14.44±7.39 |  | 0.36 |

‘^※^’ indicates the comparison between three groups, *P* > 0.05 indicates that there is no significant difference.

**4. The significant differentially expressed miRNAs (SDE-miRNAs) in SSDH and LGDH patients**

**Table S2.** The SDE-miRNAs in SSDH patients

| Up | Down | Fold Change (FC)  (SSDH *vs.* HC) | *P*  (SSDH *vs.* HC) |
| --- | --- | --- | --- |
| hsa-miR-122-5p |  | 14.77 | <0.05 |
| hsa-miR-320e |  | 3.42 | <0.05 |
| hsa-miR-1260a |  | 6.13 | <0.05 |
| hsa-miR-483-3p |  | 4.26 | <0.05 |
| hsa-miR-1273g-3p |  | 2.14 | <0.05 |
| hsa-miR-4419b  hsa-miR-451a |  | 4.88  3.17 | <0.05  <0.05 |
|  | hsa-miR-3196 | 0.38 | <0.05 |
|  | hsa-miR-223-3p | 0.37 | <0.05 |
|  | hsa-miR-15b-5p | 0.49 | <0.05 |

*P* > 0.05 indicates that there are significant differences between the two groups

**Table S3.** The SDE-miRNAs in LGDH patients

| Up | Down | Fold Change (FC)  (LGDH *vs.* HC) | *P*  (LGDH *vs.* HC) |
| --- | --- | --- | --- |
| hsa-miR-122-5p  hsa-miR-320e  hsa-miR-1260a  hsa-miR-483-3p  hsa-miR-30d-5p  hsa-miR-762 |  | 19.41  3.83  11.00  4.72  19.12  6.31 | <0.05  <0.05  <0.05  <0.05  <0.05  <0.05 |
| hsa-miR-4532  hsa-miR-22-3p |  | 7.52  4.65 | <0.05  <0.05 |
| hsa-miR-129-2-3p  hsa-miR-21-5p  hsa-miR-1304-3p  hsa-miR-129-1-3p |  | 6.66  5.50  3.74  4.58 | <0.05  <0.05  <0.05  <0.05 |

*P* > 0.05 indicates that there are significant differences between the two groups

**5. MiRNA-target gene networks built using the target genes of the SDE-miRNAs in SSDH and LGDH**

To further show the regulatory relationships between SDE-miRNAs and their target genes, the biological interaction networks were built as follows.


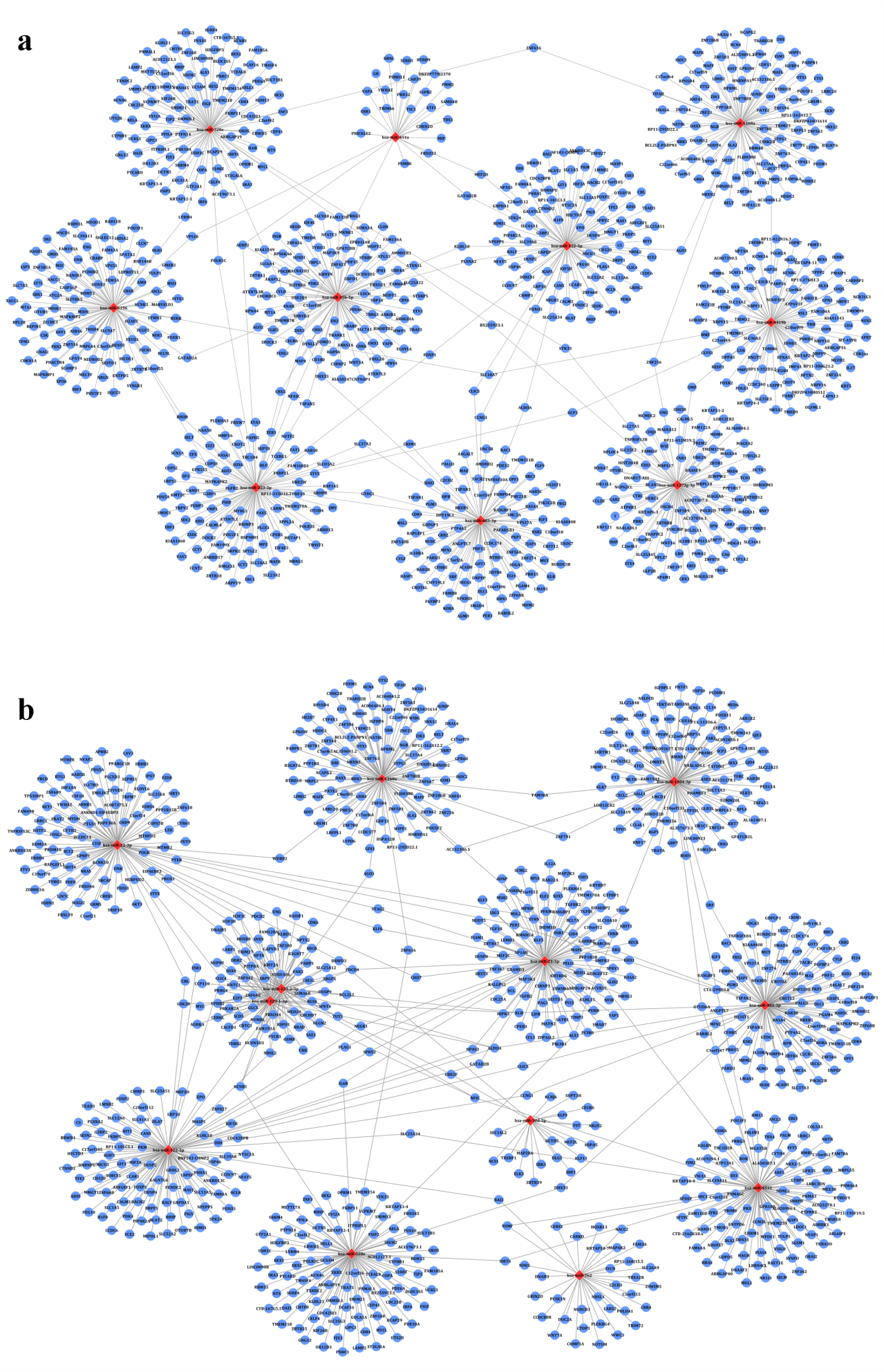


**Figure S1.** The miRNA-target gene networks built using the target genes of the SDE-miRNAs in SSDH (a) and LGDH (b). The red squares (the central nodes) represent SDE-miRNAs, and the blue spots represent the target genes. The lines indicate the interactions between SDE-miRNAs and the target genes.
